# The Bibliometric Literature on Scopus and WoS: The Medicine and Environmental Sciences Categories as Case of Study

**DOI:** 10.3390/ijerph18115851

**Published:** 2021-05-29

**Authors:** Mila Cascajares, Alfredo Alcayde, Esther Salmerón-Manzano, Francisco Manzano-Agugliaro

**Affiliations:** 1 Department of Engineering, University of Almeria, ceiA3, 04120 Almeria, Spain; milacas@ual.es (M.C.); aalcayde@ual.es (A.A.); fmanzano@ual.es (F.M.-A.); 2 Faculty of Law, Universidad Internacional de La Rioja (UNIR), Av. de la Paz, 137, 26006 Logroño, Spain

**Keywords:** bibliometry, Scopus, Web of Science, medicine, environmental science, sustainability

## Abstract

In a broad sense, science can be understood as the knowledge contained in scientific manuscripts published in scientific journals. Scientific databases index only those journals that reach certain quality standards. Therefore, research and dissemination of scientific knowledge are essential activities for the growth of science itself. The aim of this manuscript is to assess the situation of medicine and environmental sciences among the bibliometric literature and to put it in perspective with the overall bibliometric publications in all scientific fields. The main countries publishing bibliometric manuscripts are China, USA and Spain. The latter country is ranked three out of the top five institutions according to the Scopus and WoS databases. In both databases, the average scientific collaboration of the top 20 institutions offers the same result, 41%. According to Scopus, the main subject categories in which this research falls are social sciences (38%), computer science (26%) and medicine (23%), while the environmental sciences category has 8%. In the analysis of the Medicine category alone, it has been observed that 136 countries have contributions in this field. The main countries are the United States, China and the United Kingdom. In the field of medicine, the main areas studied were: Epidemiology, Pediatrics, Orthopedics, Cardiology, Neurosurgery, Radiology, Ophthalmology, Oncology, Plastic Surgery and Psychiatry. With respect to environmental sciences, less international dissemination has been found, with only 83 countries having worked in this field. The main ones are China, Spain and the United States. Regarding the top 10 institutions, it can be stated that only Spain and China are relevant. Spain focuses on sustainability and China on the environment. The result of an independent keyword analysis of all published bibliometric manuscripts has shown that the main clusters are: Mapping Science (29%), Research Productivity (23%), Medicine (20%), Environmental Sciences (12%), Psychology (7%), Nursing (6%) and Engineering (4%). In short, medicine and environmental sciences are the most relevant areas in the field of bibliometrics after social sciences and computer sciences.

## 1. Introduction

Bibliometrics, as a science-related discipline, aims to provide a set of tools for the assessment of scientific production. From its origin at the beginning of the 20th century to the present day, bibliometric studies have focused on different points of view. In 1917 Cole and Eales carried out the first bibliometric study through the statistical analysis of publications on comparative anatomy [1], thus initiating the use of bibliometrics for the measurement of scientific activity. Following this same approach, in 1926 Lotka focused his work on analyzing the scientific production of researchers with the so-called Lotka’s Law of Productivity, a law that determines that the greatest number of authors publish the least number of publications, while the least number of authors publish the greatest number of publications [2]. In 1956, Price formulated the Law of Exponential Growth of Scientific Information, stating that it grows at a much faster rate than other social processes. Price also states that the scientific literature loses relevance more rapidly, although not in a uniform manner depending on the different disciplines. Thus, while in the experimental sciences and technology the growth in number of publications is greater and faster, their decline is more rapid, in contrast to the behavior found in the humanities and social sciences. Later, it was in 1963 when Price introduced a new element in the development of bibliometrics by relating the growth of science to scientific communication [3].

A second aspect of bibliometrics is oriented to the analysis of the publications’ references in the scientific literature. Thus, in 1927 Gross and Gross made the first count of references appearing in the Journal of the American Chemical Society to study the frequency of their appearance and the sources of their origin, applying the study to the selection of the list of subscriptions of interest [4]. In 1934 Bradford analyzed the distribution of articles in journals by formulating Bradford’s Law of Dispersion, according to which it was evident that a small number of journals accounted for the largest percentage of the bibliography of a specific topic [5]. If scientific journals are arranged in decreasing order of productivity of articles on a given subject, one can distinguish a core of journals more specialized in that subject and several groups containing approximately the same core but distributed in an increasing number of journals. It can be understood as the background of the classification of journals by scientific categories.

The third point of view focuses on the analysis of the impact and visibility of research through citation activity. As early as 1873 Shepard developed a citation index following the codification applied to federal court judgments in the United States. However, it was not until 1936 that Cason and Lubotky created for the first time a citation network, identifying the links between psychology journals [6]. However, undoubtedly, the precursor of citation analysis is Garfield, who published in 1955 in the *Science* journal the proposal for a citation index [7], based on Sherpad’s concept, which made it possible to relate an article to other articles citing it. In this way it was possible to assess the significance of a research paper and its impact, and for researchers to know how their publications were being used. This is the renowned Science Citation Index (SCI) created by Garfield himself from the ISI (Institute for Scientific Information). In the early 1960s, Garfield and Sher designed the Impact Factor.

The purpose of the Impact Factor was to be the methodological instrument for selecting the journals that belong to the Science Citation Index, since it was unfeasible to include all the existing scientific journals in it. Years later, in addition to the Science Citation Index (focused on Experimental and Technological Sciences), it created the Social Science Citation Index (oriented to the Social Sciences) and the Arts and Humanities Citation Index (AHCI) for the Arts and Humanities. These three databases have been a milestone in bibliometrics and have become benchmarks in the evaluation of publications, researchers, and institutions. They are part of the Web of Science database platform, originally known as ISI Web of Knowledge and currently owned by Clarivate Analytics.

Although they have been the main benchmark since the 1960s, based also on the relationship that Garfield established in 1979 between the nature of the research and its potential to be cited, they have nevertheless been the focus of multiple criticisms [8]. Earlier in 1976 Pinski and Narin warned of the bias in favor of reviews, which tend to have a higher impact factor and in the calculation of the impact factor all citations are weighted equally [9]. To correct this deviation, they suggest the “influence methodology”, giving each journal a weight regardless of its size. As early as 1986 Tomer thought that “There is no distinction in regard to the nature and merits of the citing journals” [10]. These disagreements have been ongoing for a long time, and they are still relevant today.

For example, in 2001 Tijssen, Visser and Van Leeuwen questioned citation analysis as a measure of research quality since the influence of citation varies in different disciplines, showing considerable differences [11]. Today, shortcomings such as asymmetry between numerator and denominator, differences between disciplines, insufficient citation window and asymmetry of underlying citation distributions has also been analyzed by Larivière and Sugimoto in 2019 [12].

The JCR Impact Factor (SCI, SSCI) is not the only metric that measures the impact factor. The SJR (Scimago Journal Rank), shows the visibility of the journals contained in Scopus since 1996. This metric applies not only to journals, but also to book series and conference proceedings. Based on citations, it shows the quality and reputation of the journal in thematic fields, computing the citations received to articles of a journal for a period of three years, giving a greater weight to citations coming from high reputed journals. The SJR index attempts to correct for these deviations by weighting links based on citation proximity, extending the number of years considered in the citation and setting thresholds for self-citation within the journal itself [13].

By the end of 2016 [14], Scopus establishes a new metric index, the CiteScore, which extends the range of citation years (4 years), but by including all types of documents; on the one hand, it eliminates the differences between the different types of documents, although on the other hand some critics state that this index benefits Elsevier publications, which tend to publish a lower proportion of articles than other publishers [15].

Additionally, as a last novelty, there is the transition of the impact factor computation with respect to the date of online publication and not the date of print publication, as until now. In the current system, there are journals that have up to more than a year to publish the article online so that it can obtain citations, and when it is published in print, its number of citations is higher than those of other journals. Therefore, there is a trend towards a model in which the online publication date will be considered for the computation of the Journal Impact Factor (JIF) [16].

This change implies a problem for databases that do not have an online publication date. Web of Science Core Collection has begun to index online-first articles since December 2017 [17]. For example, in the case of Web of Science, half of the journals indexed lack this data [16]. If a publication is published online in the same year as in print, there is no mismatch since the JIF is from the same year. This is not the case for journals published online in one year and in print in another. Clarivate is considering the effects of adopting two new counting models: one pre-2020 and one post-2020 [18].

Thus far, bibliometrics has progressed from its origins to the present day. At present, there is a significant increase in the number of publications on this discipline, closely linked to the exponential growth of science. This trend has been classified into three major approaches [19]:

1. Bibliometric performance studies on authorship and production: they focus on analyzing the profiles of authors according to elements such as their affiliation, country, and the production of articles, examining which are the most cited or relevant;
2. Bibliometric studies on topics: they focus on the main topics dealt with, as well as their relationships or evolution in a specific topic;
3. Studies on research methodologies: they focus on the research methods and techniques used to develop the research papers published in the journals.

Taking all these approaches into account, how can bibliometrics be defined? From a quantitative point of view Pritchard in 1969 describes it as “studies aimed at quantifying the processes of written communication” [20]. In 1987, Broadus defined bibliometrics as the “branch of research concerned with the quantification of the physical units of publications, bibliographic citations and their surrogates” [21]. A broader concept is included here since it establishes relationships between publications and bibliographic links or co-citation. Moed in 1989 defines it as the “discipline that deals with the collection, processing and management of bibliographic data from the scientific literature” [22]. From this second point of view, bibliometrics has been defined as a tool for analysis and evaluation. In 1989 White and McCain defined it as “the quantitative study of publications as reflected in the literature, in order to provide evolutionary models of science, technology and research” [23]. Spinak in 1996 refers to bibliometrics as the study of the organization of scientific and technological sectors from bibliographic sources and patents, to identify authors, their relationships and trends [24]. In the same line, other authors describe bibliometrics as the discipline that tries to measure scientific and social activity and predict its trend by analyzing the literature [25].

Other concepts related to bibliometrics are scientometric or infometric. Scientometric applies bibliometric techniques to science and examines scientific development and policies. Infometric is more focused on quantitative aspects of measurement and the application of mathematical models.

Bibliometrics and bibliometric indexes form a whole that serve to assess and measure scientific production in all its aspects. To measure, it is necessary to evaluate a set of data that are collected in databases specialized in giving visibility to scientific publications. A bibliometric index is a parameter that measures some aspect of scientific activity and allows for assessing the impact of research in the different fields of science. The two databases that allow this analysis are Web of Science and Scopus, both with a clearly commercial bias. Based on these two databases, both Clarivate and Elsevier have developed applications that allow organizations to assess their research from different perspectives to be able to establish and evaluate strategies based on reliable data.

InCites [26] uses data from the Web of Science Core Collection since 1980 to facilitate the analysis of organizations: activity, impact, collaborations, allowing to make comparisons. It allows searching by researchers or research groups to analyze their production. The search by areas of knowledge gives an overview of emerging fields. It is also possible to analyze the journals in which they are published and the funding agencies. All these variables (affiliation, researcher, area, source of publication, funding) can be easily combined to perform analyses by applying and combining different metrics (productivity, impact, collaboration, open access) and generate all kinds of reports. As a novelty, since December 2020, InCites allows the analysis of topics, classifying them into macro, meso and micro topics thanks to the collaboration between ISI and Centre for Science and Technology Studies (CWTS) and the use of the algorithm developed by CWTS that allows to detect and connect communities [27].

Based on the analysis of data from Scopus [28], Scival offers access to more than 50 million publication records (post-1996) from over 22,000 journals from more than 5000 publishers worldwide. It analyzes the scientific output of more than 230 countries and 14,000 institutions allowing to visualize research performance, make comparisons, analyze trends, and evaluate collaborations. It also allows the analysis of topics, classifying them into topic name and topic cluster. As InCites, Scival allows to generate data analysis and visualization reports combining many metrics that assess economic impact, productivity, citation impact, usage, collaborations and communication.

There are a large number of bibliometric metrics that allow the evaluation of scientific activity, but it is important to use these metrics correctly. It is necessary to consider what is to be measured, apply the appropriate metric, detect possible deviations, make an adequate analysis, etc. In this regard the 2015 Leiden Manifesto sets out 10 basic principles that the use of metrics should not be forgotten [29], and the San Francisco Declaration on Research Assessment sets out 18 recommendations in the same direction [30].

The first goal of this research is to analyze the context of all the bibliometric studies carried out from 1996 to 2020 to discover if there is any bias towards any scientific category, if there are countries or institutions that devote a great effort to this issue and finally to analyze what consideration these works have, e.g., are they mostly considered as reviews or articles, and what level of citations they have in comparison according to the categories in which they are indexed. As a second main goal, it is the case study of the categories of medicine and environmental sciences.

## 2. Materials and Methods

This analysis was based on searches of the Scopus and Web of Science databases. A previous study has pointed out that WoS is a confusing concept, as many institutions may subscribe to only a customized subset of the entire Web of Science Core Collection. It should be made clarified that our study is conducted for the whole of WoS [31]. Although the historical content of Scopus dates to 1788, the search was limited from 1996 (when the analysis of Scopus data in SciVal began) to 2020. In the case of Web of Science, the origin of the data collected in this database begins in 1960 and the analyses in InCites begin in 1980. In order to carry a correlation in the results presented in this work, it has also been limited from 1996 to 2020.

The search was performed using the same criteria: the term “bibliometric” in the title of the publication and in the keywords assigned by the author. The results of both searches were exported from Scopus to SciVal Benchmarking and from WoS to InCites Analyze.

**Figure 1:**
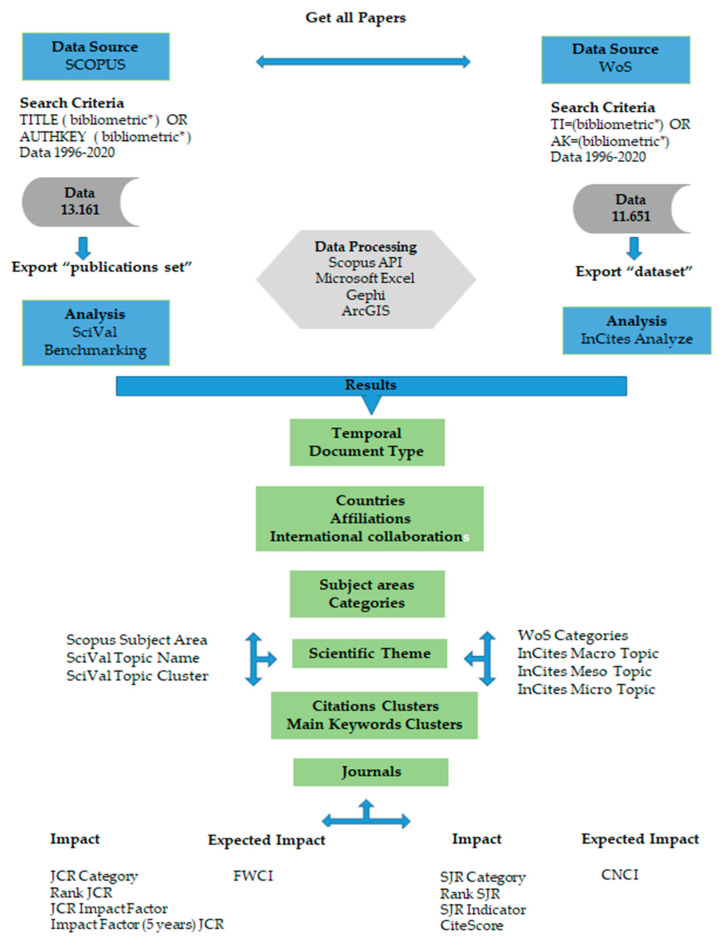
Methodology.

Data processing, both from Scopus and WoS and from SciVal and InCites, was carried out with different tools. The Scopus API was used for automatic data retrieval [32], Microsoft Excel, Gephi and ArcGIS for the analysis and representation of the results, see Figure 1.

Topic classification is done on the document [33]. A topic in SciVal covers a collection of documents with a common intellectual interest [34]. Over time, new topics appear and, as topics are dynamic, they evolve. Each document is assigned a topic consisting of three elements, for example: Intellectual Structure, Co-citation Analysis, scientometrics. The topics are based on the citation network grouping of 95% of the Scopus content (all documents published since 1996), taking as a reference the direct analysis of citations using the reference lists of the documents. As new published documents are indexed, they are added to Topics using their reference lists. This makes the Topics dynamic and most increase in size over time. New topics represent research areas that have experienced a significant acceleration of growth in recently published articles and have attracted funding. These new Topics are derived from the existing stem Topics and are formed by the new citation relationships that have occurred in the last year. Once a year, the Topics SciVal algorithm is run to identify the new Topics that have emerged [35].

Like SciVal Topics, the InCites Topics ranking is also done on the document. It is based on a CWTS algorithm [27] considering the citations (cited and citing) between documents, based on the “strength” of the citation relationships. In this way, clusters are created: macro, meso and micro topics.

An independent analysis, based on scientific communities or clusters and the relationships between them based on citation and main keywords, has also been considered in this research.

Finally, continuing with the issue of quality, the sources (journals) have been analyzed with the following metrics:

- Number of publications in WoS and Scopus;
- Number of citations in WoS and Scopus;
- Quartile in JCR and SJR;
- Journal Impact Factor JCR. It uses for the citations, articles, reviews, and proceedings papers [36];
- 5-Year Journal Impact Factor JCR, available from 2007 onward [36];
- Impact SJR [37];
- Cite Score [35].

On the other hand, the analysis of the sources has been completed with two other metric values:

- Field-Weighted Citation Impact (FWCI) the SciVal [38];
- Category Normalized Citation Impact (CNCI) the InCites [36].

## 3. Results of Bibliometric Literature on Scopus and WoS

### 3.1. Trend in Scientific Production

**Figure 2:**
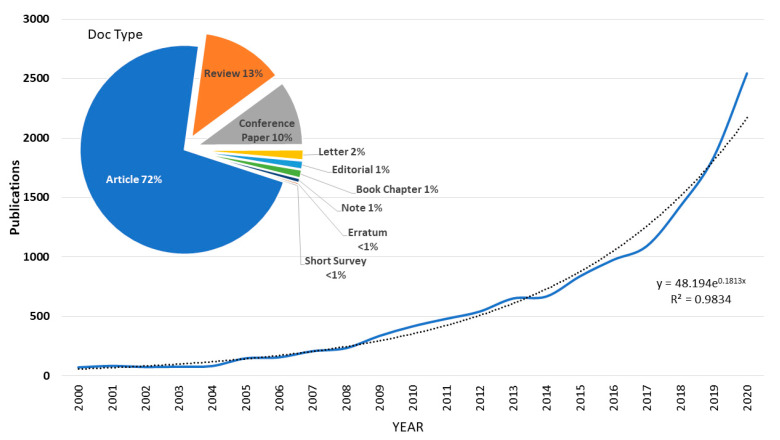
Bibliometric publications trend (Source Scopus).

According to Scopus, with the search criteria used, between 1996 and 2020, 13,161 results were obtained. The temporal evolution is shown in Figure 2 from the year 2000, since before that date there are few papers per year. The trend line has been represented, showing that the annual growth is exponential. It can be observed that in 2020 there will be more than 2500 published documents.

Figure 2 shows that 72% of the documents are mainly classified as articles. To a lesser extent, reviews in 13% of the cases and contributions to conferences in 10%. The number of reviews shows that this type of documents is the result of an analysis of a specific topic. In this case the most cited article [39] has considerably more citations than the most cited review [40].

**Figure 3:**
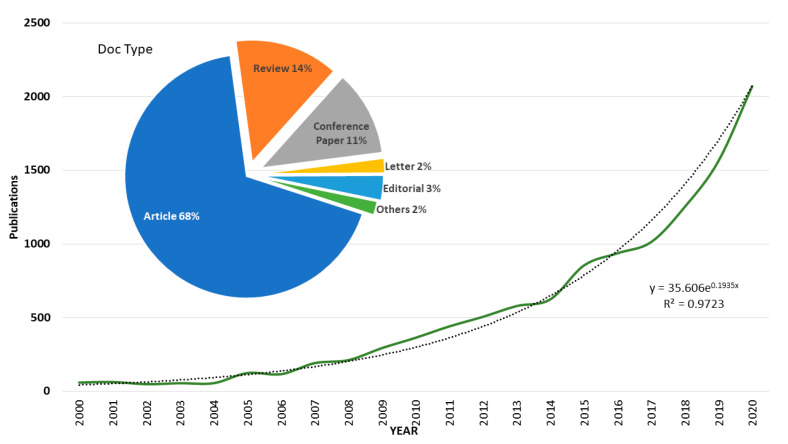
Bibliometric publications trend (Source WoS).

In Web of Science (WoS), with the same search criteria, 11,651 results were obtained between 1996 and 2020, slightly less than in Scopus. The temporal evolution is shown in Figure 3 from the year 2000, since before that date there are few papers per year, as was the case in the other database. The trend line has been plotted, showing that annual growth is exponential. It can be observed that in the year 2020 there will be more than 2000 published documents.

Figure 3 shows that 68% of the works are classified as articles. To a lesser extent, reviews in 14% of the cases and contributions to congresses in 11%. In general, there are no differences between the two databases in the distribution of documents by type. In this case the most cited article and review are the same as in Scopus.

#### 3.1.1. Countries

**Figure 4:**
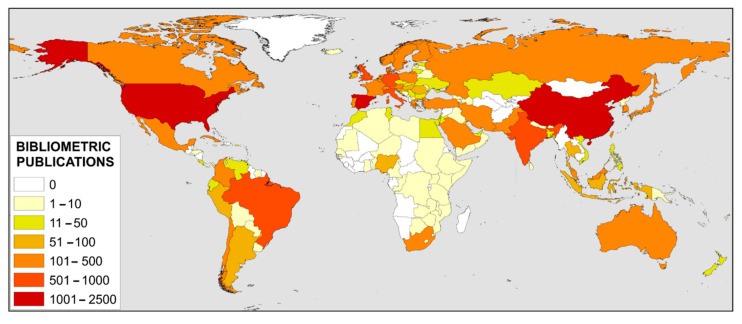
Worldwide distribution by country of scientific production on bibliometrics.

The countries that have devoted most effort to bibliometric studies are China with 16% of the total number of publications, followed by the USA with 15% and in third place Spain with 12.5%. Further behind with 6% are Brazil, the UK and India. Given that China and the USA are the world leaders in scientific production, these results in the first two positions are not surprising. It should be noted that a recent study has shown that China has overtaken the United States in terms of the number of articles indexed in the SCI in 2018 [41]. However, what is particularly notable is the great effort made by Spain in this area. Figure 4 shows a worldwide map with the geographical distribution by countries according to their publications related to bibliometrics.

The most cited bibliometric document from China is related to energy [42]. For the USA, it is the one cited above as the most cited review, and it is about economics [40], the same subject line as for the most cited from Spain [43].

#### 3.1.2. Institutions According to Scopus and WoS

Table 1 shows the top 20 institutions that publish the largest number of bibliometric publications, according to Scopus and WoS. A first analysis of the table shows that the difference between the two databases is only in four institutions. The institutions that appear in Scopus in the top 20 and are not in WoS are: An-Najah National University (18), Sichuan University (16), Universidad de Chile (14) and Universidade Federal de Santa Catarina (19). On the other hand, the four institutions that appear in WoS and not in Scopus are: Harvard University (16), University System of Georgia (13), University of London (8) and Istituto di Analisi dei Sistemi ed Informatica Antonio Ruberti (IASI-CNR) (17).

These differences are undoubtedly due to the different sources indexed in the two databases. Of the differences in this top 20, there is only one institution in the top 10 of WoS and not in Scopus, the University of London. It can be seen that the first five institutions are the same in both databases, although in different order: Universidad de Granada (Spain), University of Valencia (Spain), Consejo Superior de Investigaciones Científicas (CSIC) (Spain), Chinese Academy of Sciences (China) and Leiden University (Netherlands). It is remarkable that three institutions from Spain are in the top five, and this probably contributes, as already mentioned, to the fact that Spain accounts for 12.5% of the total number of publications in this field.

The most cited documents from these institutions were: University of Granada (Spain), related to computers and education [44]; University of Valencia (Spain), related to economics [45]; Consejo Superior de Investigaciones Científicas (CSIC) (Spain), related to bibliometrics [46]; Chinese Academy of Sciences (China), related to biodiversity and conservation [47]; and Leiden University (Netherlands), related to bibliometry, the one already reported as the most cited bibliometric article [39].

Leiden University is a benchmark in research evaluation and bibliometric studies through the Centre for Science and Technology Studies (CWTS). It works closely with Clarivate Analytics, which bases its analyses on Web of Science and is continuously expanding its data system to include other sources, such as Scopus, PubMed, Crossref, PATSTAT, Mendeley and ORCID [48].

International collaborations (IC) were analyzed for both Scopus publications using SciVal and WoS publications using InCites, see Table 1. For Scopus data, the minimum international collaboration for the top 20 is 15.8% for the Consiglio Nazionale delle Ricerche (CNR), while the maximum is 81% for the Universidad de Chile. For WoS data, the minimum of international collaboration in this top 20 is 10% from Istituto di Analisi dei Sistemi ed Informatica Antonio Ruberti (IASI-CNR); while the maximum is 79.5% from Georgia Institute of Technology. However, both databases, for the average scientific collaboration of this top 20 offer the same result, 41.4% according to Scopus and 41% according to WoS. The first five institutions have relatively low international scientific collaboration in this field, between 21 and 38%. However, if we analyze the average of these five institutions, it is 29.8% according to Scopus and 29.9% according to WoS. Therefore, it is possible to establish that the main institutions dedicated to bibliometrics collaborate less than the average of the other 15, which without them have an average of 45% of international collaboration in both databases.

### 3.2. Scientific Areas of Indexing

#### 3.2.1. Scopus

##### Subject Area

**Figure 5:**
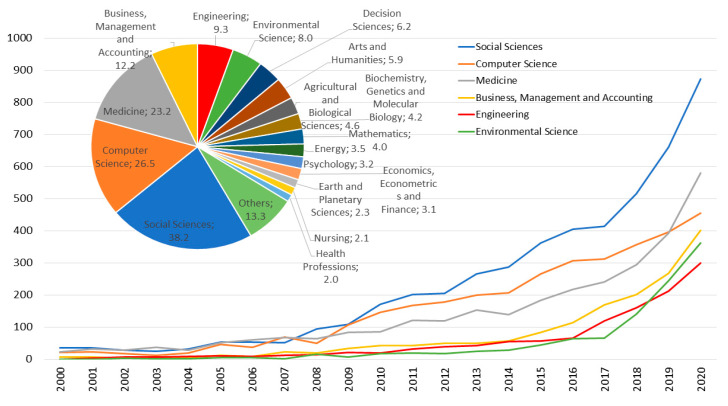
Subject area in Scopus and its trend from 2000 to 2020.

Figure 5 shows the indexation by subject area in Scopus. The Social Sciences category leads the published documents with slightly more than 38% of the publications, which was to be expected since this is where bibliometrics is classified. In second place is the Computer Science category with 26.5%, showing that there is an increasingly important volume of data management and that therefore advanced computer techniques must be applied. The third category in order of number of documents is the field of Medicine with more than 23%, this is worth a reflection on the importance of bibliometrics. The next three categories are close to 10% and are: Business, Management and Accounting (12%), Engineering (9%) and Environmental Science (8%).

Figure 5 shows the temporal evolution by years of the first six categories from 2000 to 2020 according to Scopus. Since 2008, bibliometric publications have been led by the Social Sciences category. The Computer Science category has occupied the second place from 2009 to 2019, and already in the last year it is surpassed by the Medicine category, which was in third place since 2009. The next three categories have had a quite similar behavior, exceeding 100 publications per year the Business, Management and Accounting category in 2016, Engineering in 2017 and Environmental Science in 2018, all of them finish with 300 or more papers per year in the last year studied, 2020.

##### SciVal

According to SciVal, the average number of citations per document was 12.4. This section starts to discuss the Topic Name extracted from Scival, see Table 2. It is observed that the main topic name is Hirsch Index, Self-Citation, Journal Impact Factor; followed closely by: Intellectual Structure, Co-citation Analysis, scientometrics. In third place is: Co-Authorship, Scientific Collaboration, scientometrics.

Since the Hirsch Index or H index was proposed in 2005 [49], many evaluation agencies and even journals make use of it to measure the quality of an individual author’s impact. This has also given rise to the misconduct by some authors of self-citation to artificially raise their own H index [50]. There are studies that propose eliminating self-citation for the calculation or correction of the H index [51]. Self-citations do not only occur in individual authors, but some journals have been able to encourage this practice in citing articles from their own journal to raise its Journal Impact Factor [52], this is named journal self-citation. These facts have inspired many studies that make this Topic Name the most prominent one to date.

In the second topic name, these studies are based on describing the intellectual structure of a particular scientific field from the point of view of frequently occurring keywords and phrases, using Co-citation Analysis, co-word analysis, hierarchical clustering, and link analysis [53]. The third of the main topic name focuses on the analysis of the structure of scientific collaboration networks [54]. These scientific collaboration networks are analyzed by scientific fields [55], countries [56,57] or even institutions [58,59,60].

Table 2 lists each topic name according to the average number of citations received per document. According to this index, the leading topic name is Social Science and Humanities, Research Evaluation, Book Publishers with almost 45 citations per document, followed in second place by Technology Roadmapping, Patent Analysis, Technological Competitiveness with almost 23, and in third place by Bibliometric Analysis, Citation Index, Document Type with almost 19.

Table 3 shows the main topic clusters related to bibliometric studies. The main topic cluster is the one focused on: Publications, Periodicals as Topic, Research. This cluster stands out from the rest as it is 11 times larger than the next cluster, which is focused on: Industry, Innovation, Entrepreneurship; and 30 times larger than the third: Library, Librarian, Information. In relation to the citations of each topic cluster name, Decision Making, Fuzzy Sets, Models leads this ranking with 23 citations per document, e.g., the manuscript “Fuzzy decision making: A bibliometric-based review” [61] has 163 citations according to Scopus. In second place is: Industry, Innovation, Entrepreneurship with 18 citations per document. In third place is Electricity, Energy, Economics with 16 citations per document, e.g., “Power quality: Scientific collaboration networks and research trends” [62].

#### 3.2.2. WoS

##### Categories

The classification by WoS categories is shown in Table 4. As is well known, the categories do not match those of Scopus. On the other hand, in both databases the same document can be indexed in more than one category if the journal in which it was published is indexed in more than one category. For the documents analyzed, the great discrepancy between scientific fields between the two databases is observed in the field of Medicine in Scopus, which does not correspond to the first positions ranked by WoS. Although there are comparable categories in WoS such as: Medicine, Research and Experimental or Medicine, General and Internal, there are many other categories specific to the medical field that are independent for indexing. In our case, for example, the categories of: Oncology, Psychiatry, Pediatrics, Anesthesiology, Respiratory System, Ophthalmology, Dermatology or Tropical Medicine, but all of them with values below 1%, which does not make it possible to reach the 23.2% that appeared in Scopus. Therefore, the indexing field of medicine is very different between the two databases.

In the last column of Table 4, the average number of citations of these bibliometric documents has been calculated according to WoS data. For the whole documents analyzed the average number of citations per document was 11.7. Only three categories are below five citations per document: Engineering, Electrical and Electronic, Computer Science, Theory and Methods and Social Sciences, Interdisciplinary. In general, these documents are highly cited within their scientific categories, especially in Management and Engineering, Industrial, both with more than 18 citations per document (C/D).

##### Incites

In this section the macro, meso and micro topics in which WoS classifies all bibliometric publications will be discussed. The macro topics are listed in Table 5. Leading this classification are the social sciences which has 5 times more documents than the following one. Followed by Clinical and Life Sciences, and in third place is Electrical Engineering, Electronics and Computer Science, with far fewer documents.

In terms of citations per document, social sciences remain the main one with 14. However, now the second place in this other ranking is for Electrical Engineering, Electronics and Computer Science with 12 citations per document. With 10 citations per document there are already several categories: Chemistry, and Engineering and Materials Science. The average number of citations per document (C/D) is 8.5.

The 20 main meso topics are listed in Table 6, highlighting bibliometrics, scientometrics and Research Integrity, with 11 times more publications than the second meso topic, Management. These two meso topics can be included within the main macro topic of Social Science, mentioned above. As can be seen in column 2 of Table 6, the first number indicates the macro topic. It can be observed that in this top 20 are not present the macro topics of: Chemistry (2), Earth Sciences (8), Engineering and Materials Science (7), Arts and Humanities (10), Physics (5) or Mathematics (9).

The two meso topics with the most citations per document are Artificial Intelligence and Machine Learning (19 C/D), Operations Research and Management Science (17 C/D), both from the macro topic 4, Electrical Engineering, Electronics and Computer Science. The average number of citations per document for this top 20 meso topic is 11.7 C/D.

Finally, the micro topics, as expected, the first one, bibliometrics, belongs to the bibliometrics, scientometrics and Research Integrity meso topic, see Table 7. Additionally, the second, Knowledge Management, and the fourth, Corporate Social Responsibility, belong to the Management meso topic. The third, Systematic Reviews, is included in the Medical Ethics meso topic. In the first 20 micro topics there is an average of 15 C/D. Fuzzy Sets stands out above all with more than 30 C/D and belongs to the meso topic with the highest average number of citations per document, Artificial Intelligence and Machine Learning.

### 3.3. Source (Journal)

Table 8 shows the top 20 journals indexed in both WoS and Scopus, and where the bibliometric articles are published. The table shows both the ranking of the journal by total number of publications in the subject studied and by citations received for these articles. In addition, the different impact indicators according to JCR, SJR and Scopus and the relative position of the journal within its category according to JCR and SJR, e.g., the quartile, are also shown.

The first consideration for journals is that they should have not the same number of articles published in the same period in both databases. What probably happens is that editorial articles or short communications are considered differently in both databases.

It is noted that apart from the journals indexed in the category of Information Science and Library Science, there are many of them in the categories of Environmental Sciences Environmental Studies such as: *Sustainability*, *Journal of Cleaner Production*, Environmental *Science* and *Pollution Research*. Or even Journals in the field of Medicine such as *Medicine* or *World Neurosurgery*.

Considering the quartile of the journals, it can be found that according to JCR: six are Q1, six are Q2, five are Q3, two are Q4 and one does not have a JCR impact factor. That is to say that most are Q1 and Q2. According to Scopus: seven are Q1, nine are Q2, one Q3 and three have no SJR. Of all these journals, the one with the highest impact both IF JCR and SJR is *Journal of Informetrics*.

A comparative study of the top 10 countries and affiliations publishing in the leading bibliometrics journal, *Scientometrics*, is shown in Table 9. If the results obtained in Table 9 are compared with the global results of scientific production by country, it can be seen that the first three countries are the same and in the same ranking order: China, the United States, and Spain. Another four countries that appear in the top 10 of both rankings, although in a different order, are: United Kingdom, Germany, India and Italy. In summary there is an overlap of 7 of the 10 countries in both rankings. Although China and the USA are the two countries with the most publications, the Netherlands dominates in citations per document with 22 followed by Hungary with 19.

With regard to affiliations, something similar happens, since of the top 10 that publish the most in *Scientometrics*, 6 are in the top 20 worldwide. These are: Universidad de Granada, Consejo Superior de Investigaciones Científicas (CSIC), Chinese Academy of Sciences, Leiden University, Wuhan University and KU Leuven. In the case of the affiliations, i.e., the most productive ones are also the most cited in *Scientometrics* journal: KU Leuven (18 C/D), Magyar Tudomanyos Akademia (21 C/D) and Leiden University (35 C/D).

### 3.4. CNCI vs. FWCI

Table 10 shows the CNCI and FWCI. Both the CNCI and the FWCI measure the actual citation impact on the expected citation for the articles studied. As long as it is equal to or greater than 1, they have achieved the expected citation. There are only three journals that in both indicators, CNCI and FWCI, are below one: *Current Science*, *Malaysian Journal of Library*, and *Information Science*, and *Revista Española de Documentación Científica*. Then, there are two that have a CNCI < 1, although the FWCI is above 1: *Sustainability*, and *Environmental Science and Pollution Research*. All the other journals, 15 out of 20, are above 1 in both indicators, so in general the bibliometric articles achieve a higher number of citations than expected based on the journal and category.

Considering the number of citations per document, for Incites the average is 15.5, and for Scival it is 14.8, so that for this select group of journals the average is about 15. The three journals with the most citations per document according to Incites are: *Research Policy* (62 C/D), *Technological Forecasting and Social Change* (31.7 C/D) and *Journal of Informetrics* (28 C/D). The lowest one for Incites is *Investigación Bibliotecológica* (0.9 C/D). The three journals with the most citations per document according to Scival are: *Journal of the American Society for Information Science and Technology* (48.4 C/D), *Technological Forecasting and Social Change* (42.9 C/D) and *Journal of Informetrics* (37 C/D). The lowest one for Scival is *Espacios* (0.7 C/D).

**Figure 6:**
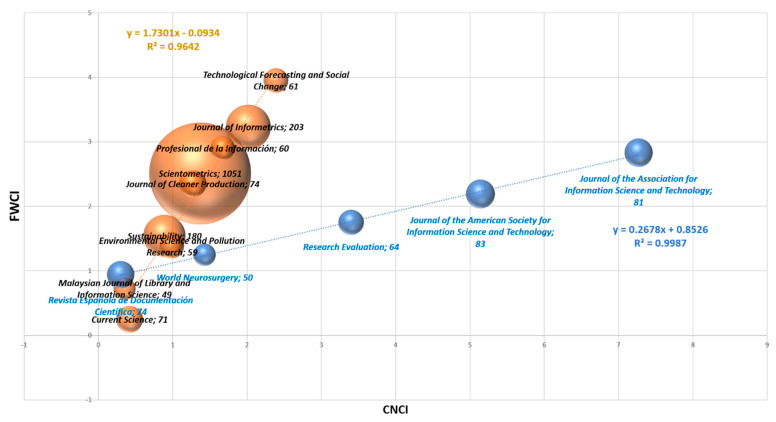
CNCI vs. FWCI for the top 20 journals.

Figure 6 shows the journals studied in Table 11, where the size of the dot is the number of articles studied. Both indicators, FCWI and CNCI, have been plotted, here two trends have been observed. The first one involving the largest number of journals is slightly favored by the FWCI. The second trend, which favors CNCI over FWCI, occurs in the journals: *Journal of the American Society for Information Science and Technology*, *Research Evaluation*, *Journal of the Association for Information Science and Technology*, *World Neurosurgery*, and *Revista Española de Documentación Científica*.

## 4. The Medicine and Environmental Sciences Categories as Case of Study

Once all the bibliometric manuscripts have been analyzed, it has been observed that the two main categories are those that could be classified as natural for bibliometrics, the social sciences and computer sciences. After these, the third category has been found to be medicine, and the other emerging category is environmental sciences. These two categories are therefore worth studying as a case study, which is the second objective of this manuscript.

### 4.1. The Medicine Category

#### 4.1.1. Countries and Affiliations

**Figure 7:**
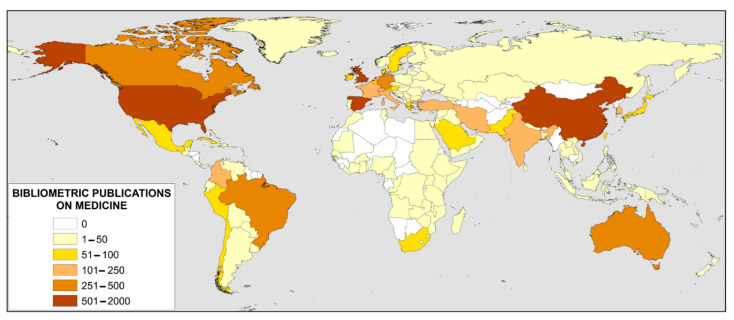
Global distribution of bibliometric publications by country in the medicine category.

Figure 7 shows a worldwide map with the distribution by country of bibliometric publications in the medicine category. Publications from 136 different countries have been found. It can be seen that it covers geographically all the countries of the world.

Table 11 shows the top 10 countries and affiliations publishing on bibliometrics in the category of medicine. They have been analyzed from 2000 to 2020 and based on the Scopus database.

In terms of countries, this ranking is led by the USA with more than twice more publications than the next country, China. It should be noted that the most cited article from the USA in this category is on the history and meaning of the impact factor, even though it is published in a medical journal, the *Journal of the American Medical Association* (JAMA) [63]. Although the second most cited manuscript from this country is on the effectiveness of interventions, whose results are subsequently contradicted [64].

In third place is the UK where its most cited manuscript is related to a taxonomy of behavior change techniques used in interventions [65]. For the fourth country, Spain, the most cited manuscript can also be considered a bibliometric research paper related exclusively to medicine, the Spanish version of the Short Form 36 Health Survey [66].

Among the top 10 affiliations that have published bibliometric manuscripts in the category of medicine, there are three from Spain, University of Valencia, Consejo Superior de Investigaciones Científicas and Universidad Miguel Hernandez de Elche; and the other three from Canada: University of Toronto, McMaster University and The University of British Columbia. The two most cited manuscripts from the University of Valencia focus on bibliometric aspects of scientific collaborations [67], or the impact factors of medical journals [68] and the third most cited manuscript focuses on a purely medical topic with the leishmaniasis [69]. The most cited manuscript from the University of Toronto is a purely medical one, such as the propensity-score methods that are increasingly being used to reduce the impact of treatment-selection bias in the estimation of treatment effects using observational data [70].

#### 4.1.2. Keywords

In this section the most frequent keywords in the fields of medicine that appear in the bibliometric publications in this category have been identified, see Table 12. Among the scientific fields of medicine, Epidemiology and Pediatrics stand out above the rest. The main affiliations in these two fields are Universidad Tecnológica de Pereira (Colombia) and University of Valencia (Spain), respectively.

#### 4.1.3. Journals

Table 13 shows the top 10 journals publishing articles in bibliometrics in the category of medicine and their main WoS-JCR and Scopus-SJR bibliometric source indices. It can be seen that the top three journals are above 80 manuscripts and stand out from the rest. Of these 10 JCR journals, three are Q1, three Q2, three Q3 and one has no impact factor. However, for SJR, five are Q1, four Q2 and one Q3.

### 4.2. The Environmental Sciences Category

#### 4.2.1. Countries and Affiliations

**Figure 8:**
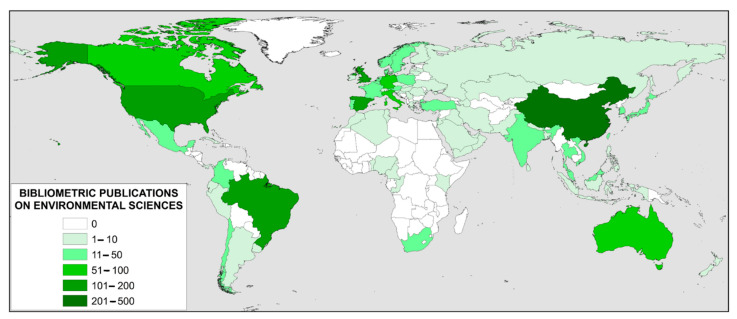
Global distribution of bibliometric publications by country in the environmental sciences category.

Figure 8 shows a world map with the country distribution of bibliometric publications in the environmental sciences category. Publications from 83 different countries have been found. It can be seen that it covers geographically a large part of the world, and that Africa is the continent with the fewest publications in this regard.

Table 14 shows the top 10 countries and affiliations publishing on bibliometrics in the category of Environmental Sciences. They have been analyzed from 2000 to 2020 and based on the Scopus database. By country, this ranking is led by China, with more than twice as many publications as the next country, Spain. Notably, the most cited article from China in this category is on sustainable, smart, resilient and low-carbon cities [71]. The second most cited manuscript from this country is on anaerobic digestion of food waste [72].

Number two in this category, Spain, has its most cited article on sensitivity analysis in chemical modelling [73]. The following is on green innovation [74]. Number 3 in this category, USA, has its most cited article on urban resilience [75]. The following are on scholarly networks on resilience, vulnerability and adaptation within the human dimensions of global environmental change [76]. Impacts of anthropogenic noise on marine life [77].

Among the top 12 affiliations that have published bibliometric manuscripts in the environmental sciences category, there are 10 from China and 2 from Spain. The top two affiliations are the Chinese Academy of Sciences and the University of Almeria. The two most cited manuscripts from the Chinese Academy of Sciences are related to global biodiversity [47] and, the other on ecological engineering and ecosystem restoration [78]. For the University of Almeria, the most cited manuscripts are related to and nitrate leaching [79] and energy efficiency in public buildings [80].

#### 4.2.2. Keywords

In this section the most frequent keywords in the fields of environmental sciences that appear in the bibliometric publications in this category have been identified, Table 15. Among the scientific fields of environmental sciences, sustainability and sustainable development keywords stand out above the rest. The two main affiliations for these top 10 keywords, are the University of Almeria (Spain) and the Chinese Academy of Sciences (China). The third main affiliation is the Goethe-Universität Frankfurt am Main (Germany) and the environmental topic is related to public health.

#### 4.2.3. Journals

Table 16 shows the top 10 journals publishing articles in bibliometrics in the category of environmental science and their main WoS-JCR and Scopus-SJR bibliometric source indices. It can be seen that the top journal is *Sustainability* with a large number of bibliometric manuscripts. The second and third journals are *Journal Of Cleaner Production* and *International Journal Of Environmental Research And Public Health*, respectively. Among these 10 JCR journals, four are Q1, three Q2, one Q3 and two have no impact factor. However, for SJR, five are Q1, three Q2 and two Q3.

## 5. Independent Cluster Analysis of Bibliometric Publications

In this section, all the papers have been classified by analysis of scientific communities or clusters, and their links between them, by means of the citations they make to each other. Afterwards, the most frequent keywords have been extracted from each of these scientific communities to name them, see Table 17. Bibliometrics and Bibliometric Analysis are the search terms and excluded.

**Figure 9:**
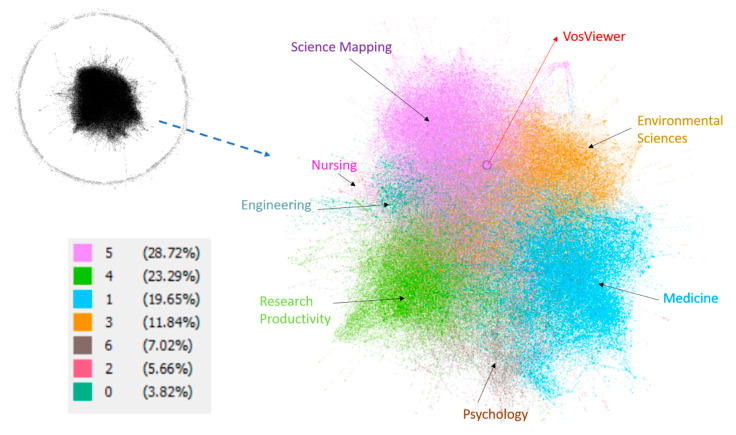
Scientific communities of bibliometric publications.

Figure 9 shows the graph generated with all the articles, where in the outer circle are documents not related to any other, or in other words, documents that do not cite any other bibliometric work, and therefore are in a certain way isolated from the core of the bibliometric publications. On the other hand, the central core are papers related by references, since they cite each other and thus establish a relationship. From this core of publications, seven communities or clusters have been detected, which are represented by colors in Figure 9. In this figure, a particular paper has also been marked in red, which is the most cited article by all the bibliometric papers (Van Eck and Waltman, 2010).

The clusters have been outlined in Table 8, where the 20 main keywords have also been collected. These clusters are: Science Mapping (28.72%), Research Productivity (23.29%), Medical research (19.65%), Environment (11.84%), Psychology (7.02%), Nursing (5.66%) and Engineering (3.82%).

Table 18 shows, for each cluster, the use of WoS or Scopus, being mainly highlighted in the Environment cluster. The only exception to this is in the Nursing cluster, where Scopus is preferred.

## 6. Conclusions

This study has analyzed the bibliometric documents produced between 1996 and 2020. It has been observed how bibliometrics were applied to research in all scientific fields during these years. To evaluate these documents, a methodology has been used that has proven to be valid to relate scientific production in Scopus and WoS and link it to bibliometric indicators through SciVal and InCites.

The first conclusion drawn from this work is that there is an exponential growth in publications between 2000 and 2020 and that most of the documents are indexed as articles (72% in Scopus and 68% in WoS), as opposed to reviews (13% in Scopus and 14% in WoS). Three countries have led the number of documents published: China with 16%, the USA with 15% and in third place Spain with 12.5%. In this sense, it is worth highlighting the role of Spain in third place compared to the two large countries with the highest scientific production in absolute terms.

From the point of view of the institutions, there are differences between the two databases analyzed. However, the top five positions in the ranking are shared by the same institutions: University of Granada, University of Valencia, Consejo Superior de Investigaciones Científicas (CSIC), Chinese Academy of Sciences and Leiden University. Once again, the predominance of Spanish institutions in this ranking stands out. International collaboration is undoubtedly a parameter that allows us to know the synergies in scientific production. In this case it has been shown that the institutions located in the top five positions of the ranking do not have a parallelism between quantity of production and international collaboration, they have 30% of international collaboration, that is to say, they have collaboration below the average, which without these institutions is 45%.

Regarding the topics where bibliometrics is applied, the publications have been categorized, and despite the differences between Scopus and WoS when classifying the publications, the results show that this type of studies have been classified mainly in the areas most related to bibliometrics. According to Scopus, in order of importance: Social Science and Computer Sciences, Medicine, Business, Management and Accounting, Engineering and Environmental Science. According to WoS: Information Science and Library Science, Computer Science, Environmental Sciences and Management. There is a high degree of interest in the application of bibliometrics to other disciplines as an element of analysis of their own progress.

Completing the review of the topics, the topics for Scopus indexing have been considered as an indicator of where the publications on bibliometrics stand out. In this sense, the trend also shows the predominance of topics related to the discipline addressed in this research. Hirsch Index, Self-Citation and Journal Impact Factor as predominant Topic Name in SciVal. Publications, Periodicals as Topic, Research as predominant Topic Cluster Names. Interestingly, the ones with the most citations per document are for the Topic Name, Social Science and Humanities, Research Evaluation and Book Publishers with 45 citations per document as average; and for the Topic Cluster Name, Decision Making, Fuzzy Sets, Models with 23 Cites per Document.

In InCites they are mostly included in the Macro Topic of Social Sciences with an average of 14 citations per document, in the Meso Topic of bibliometrics, scientometrics and Research Integrity, but with respect to citations per document the meso topic of Artificial Intelligence and Machine Learning stands out (19 C/D). In the Micro Topic, the main one by number of documents is bibliometrics, but regarding citations per document Fuzzy Sets stands out above all with more than 30 C/D. That is to say that in the citations per document the computer science topics stand out.

The analysis of the sources shows that, despite the different indexing criteria of JCR and SJR, there is variety in the categories in which they have been indexed. The first positions, according to the number of publications, are occupied by journals specialized in bibliometrics, but journals specialized in Medicine or Environment also appear among the first 20 journals. In terms of quartile ranking, a greater number of SJR journals are positioned in Q1 and Q2 compared to JCR, undoubtedly due to the different indexing criteria applied by the two databases. To complete the quartile ranking, impact factors and citation level, two metrics have been used that allow the performance of the sources based on the citations received and those expected to be received. The InCites CNCI shows that 7 of the 20 are below 1 and the SciVal FWCI shows that 9 of the 20 are also below this threshold.

In the analysis of the Medicine category alone, it has been observed that 136 countries have contributions in this field. The main countries are the United States, China and the United Kingdom. In the field of medicine, the main research areas studied were: Epidemiology, Pediatrics, Orthopedics, Cardiology, Neurosurgery, Radiology, Ophthalmology, Oncology, Plastic Surgery and Psychiatry.

With respect to Environmental Sciences category, less international dissemination has been found, with only 83 countries having worked in this field. The main ones are China, Spain and the United States. Regarding the top 10 institutions, it can be stated that only Spain and China are relevant. Spain focuses on sustainability and China on the environment. In the field of Environmental Science, the main research areas studied were: Sustainability, Sustainable Development, Climate Change, Ecology, Environmental Impact, Biodiversity, Environmental Protection, Environmental Management, Public Health and Environmental Monitoring.

The relationships between the citations of the publications have allowed, with an independent analysis, to establish clusters by key words based on the level of citation. These seven clusters were: Science Mapping, Research Productivity, Medicine, Environmental Sciences, Psychology, Nursing and Engineering. In the seven communities in which the 20 main keywords were collected, a predominance of terms related to bibliometrics applied to the different clusters was again observed. The main country keyword data has also been extracted, highlighting the relevance of China as the predominant country in four of the seven clusters analyzed. The independent analysis of the indexing category of the journals highlights that Medicine and Environmental Sciences are the most relevant areas in the field of bibliometrics, after Social Sciences and Computer Science.

In conclusion, there are many parameters that can be used to see the evolution of bibliometric studies in the period under analysis. In this case, bibliometric data and indicators have been used to study the evolution of this discipline over the years and the performance of publications. In any analysis it is important to start from the objectives of the study to be able to apply the appropriate metric values. In this sense, the recommendations established in the Leiden Manifesto and the San Francisco Declaration should not be forgotten to make proper use of the metrics that allow scientific production to be correctly assessed.

## Figures and Tables

**Table 1:** Main affiliations according to Scopus and WoS.

Rank	Scopus				WoS			
	Affiliation	N_TOT_	N_IC_ SciVal	IC (%)	Affiliation	N_TOT_	N_IC_ InCites	IC (%)
1	Universidad de Granada	259	55	21.2	Consejo Superior de Investigaciones Científicas (CSIC)	198	47	23.7
2	University of Valencia	211	74	35.1	University of Granada	198	49	24.7
3	Consejo Superior de Investigaciones Científicas (CSIC)	196	61	31.1	Leiden University	154	59	38.3
4	Chinese Academy of Sciences	188	50	26.6	Chinese Academy of Sciences	138	46	33.3
5	Leiden University	177	62	35.0	University of Valencia	133	39	29.3
6	Universidade de São Paulo	136	22	16.2	Asia University Taiwan	129	83	64.3
7	Asia University Taiwan	133	91	68.4	Max Planck Society	120	57	47.5
8	Wuhan University	118	39	33.1	University of London	115	67	58.3
9	Consiglio Nazionale delle Ricerche (CNR)	114	18	15.8	Consiglio Nazionale delle Ricerche (CNR)	112	20	17.9
10	Peking University	111	61	55.0	University of Rome Tor Vergata	109	18	16.5
11	University of Rome Tor Vergata	109	18	16.5	Peking University	101	53	52.5
12	Administrative Headquarters of the Max Planck Society	106	52	49.1	Wuhan University	101	34	33.7
13	Universitat Politècnica de València	104	35	33.7	University System of Georgia	90	67	74.4
14	Universidad de Chile	100	81	81.0	KU Leuven	80	59	73.8
15	KU Leuven	92	62	67.4	Universitat Politecnica de Valencia	80	30	37.5
16	Sichuan University	85	36	42.4	Harvard University	78	35	44.9
17	Georgia Institute of Technology	85	65	76.5	Istituto di Analisi dei Sistemi ed Informatica Antonio Ruberti (IASI-CNR)	78	8	10.3
18	An-Najah National University	85	26	30.6	Georgia Institute of Technology	73	58	79.5
19	Universidade Federal de Santa Catarina	82	19	23.2	University of Barcelona	73	34	46.6
20	Universitat de Barcelona	80	56	70.0	Universidade de São Paulo	72	9	12.5

N_TOT_ = Total number of publications; N_IC_ = number publications with international collaboration.

**Table 2:** Topic Name (Scival) for bibliometrics publications.

Topic Name	N	C	C/D
Hirsch Index, Self-Citation, Journal Impact Factor	1005	16,417	16.34
Intellectual Structure, Co-citation Analysis, Scientometrics	980	17,639	18.00
Co-Authorship, Scientific Collaboration, Scientometrics	743	11,159	15.02
Citation Counts, Bibliometric Analysis, Journal Impact Factor	438	4897	11.18
Scientometrics, Research Productivity, Bibliometric Analysis	319	1580	4.95
European Regional Development Fund, Bibliometric Indicators, ERDF	283	2186	7.72
Beauties, Citations, Sleeping Beauty	220	2295	10.43
Social Science and Humanities, Research Evaluation, Book Publishers	198	8895	44.92
Bibliometric Analysis, Citation Index, Document Type	188	3472	18.47
Readership, Citation Counts, Journal Impact Factor	186	2863	15.39
Scientific Journals, Doctoral Thesis, Spanish Universities	146	1078	7.38
Technology Roadmapping, Patent Analysis, Technological Competitiveness	145	3306	22.80
Female Scientist, Research Productivity, Women in Science	120	1596	13.30
Research Productivity, Bibliometric Analysis, Arab Countries	114	1213	10.64
Scientific Publications, Research Productivity, Bibliometric Analysis	101	1090	10.79
Tourism Research, Tourism and Hospitality, Hospitality Management	85	1517	17.85
Citations, Summarization, Scholarly Publication	68	647	9.51
Open Access Publishing, Scholarly Communication, Preprints	67	586	8.75
Economists, Co-Authorship, Economic Journals	61	596	9.77
Library Science, Tenure, Land Information System	57	495	8.68

N = Total number of publications; C = total number of citations; C/D = cites per document.

**Table 3:** Topic Cluster Name (Scival) for bibliometrics publications.

Topic Cluster Name	N	C	C/D
Publications, Periodicals as Topic, Research	6020	84,217	13.99
Industry, Innovation, Entrepreneurship	536	9593	17.90
Library, Librarian, Information	196	1560	7.96
Research, Meta-Analysis as Topic, Guidelines as Topic	163	1530	9.39
Periodicals as Topic, Open Access, Library	146	1560	10.68
Tourism, Tourists, Destination	133	1987	14.94
Industry, Research, Marketing	130	1429	10.99
Supply Chains, Supply Chain Management, Industry	129	1907	14.78
Semantics, Models, Recommender Systems	114	1171	10.27
Corporate Social Responsibility, Corporate Governance, Firms	110	1277	11.61
Schools, Brazil, Education	108	382	3.54
Electricity, Energy, Economics	101	1605	15.89
Brazil, Health, Nursing	95	363	3.82
Libraries, Metadata, Ontology	81	246	3.04
Work, Personality, Psychology	78	911	11.68
Students, Medical Students, Education	77	563	7.31
Construction, Construction Industry, Project Management	74	971	13.12
Research, Data, Information Dissemination	60	676	11.27
Rotavirus, Norovirus, Coronavirus	56	369	6.59
Decision Making, Fuzzy Sets, Models	51	1184	23.22

N = Total number of publications; C = total number of citations; C/D = cites per document.

**Table 4:** Indexing by category according to WoS.

Category	N	%	C/D
Information Science and Library Science	2508	16.3	15.8
Computer Science, Interdisciplinary Applications	1552	10.1	17.7
Computer Science, Information Systems	666	4.3	14.3
Environmental Sciences	616	4.0	9.5
Management	521	3.4	18.2
Business	379	2.5	16.8
Public, Environmental and Occupational Health	373	2.4	7.7
Green and Sustainable Science and Technology	331	2.1	12.0
Surgery	299	1.9	8.5
Environmental Studies	289	1.9	8.7
Education and Educational Research	270	1.8	5.0
Economics	225	1.5	5.4
Clinical Neurology	204	1.3	9.2
Computer Science, Theory and Methods	195	1.3	2.6
Computer Science, Artificial Intelligence	194	1.3	9.9
Engineering, Electrical and Electronic	174	1.1	4.4
Operations Research and Management Science	171	1.1	17.1
Health Care Sciences and Services	165	1.1	11.0
Social Sciences, Interdisciplinary	162	1.1	3.5
Engineering, Industrial	145	0.9	18.1

N = Total number of publications; C/D = cites per document.

**Table 5:** Macro topics (InCites).

Macro Topic	Code	N	C	C/D
Social Sciences	6	5614	80,783	14.39
Clinical and Life Sciences	1	1047	7771	7.42
Electrical Engineering, Electronics and Computer Science	4	387	4732	12.23
Agriculture, Environment and Ecology	3	278	2587	9.31
Chemistry	2	105	1068	10.17
Earth Sciences	8	62	522	8.42
Engineering and Materials Science	7	46	500	10.87
Arts and Humanities	10	44	199	4.52
Physics	5	29	83	2.86
Mathematics	9	14	68	4.86

N = Total number of publications; C = total number of citations; C/D = cites per document.

**Table 6:** Meso topics (InCites).

Meso Topic	Code	N	C	C/D
Bibliometrics, Scientometrics and Research Integrity	6.238	4489	67,420	15.02
Management	6.3	397	6049	15.24
Medical Ethics	1.155	144	1477	10.26
Sustainability Science	6.115	114	1633	14.32
Nursing	1.14	101	808	8.00
Knowledge Engineering and Representation	4.48	90	484	5.38
Education and Educational Research	6.11	86	716	8.33
Hospitality, Leisure, Sport and Tourism	6.223	70	1053	15.04
Forestry	3.40	69	853	12.36
Healthcare Policy	1.156	58	569	9.81
Economics	6.10	57	595	10.44
Climate Change	6.153	56	511	9.13
Artificial Intelligence and Machine Learning	4.61	51	1012	19.84
Human Geography	6.86	48	559	11.65
Design and Manufacturing	4.224	47	715	15.21
Social Psychology	6.73	41	247	6.02
Operations Research and Management Science	6.294	40	691	17.28
Supply Chain and Logistics	4.84	37	581	15.70
Marine Biology	3.2	35	215	6.14
Psychiatry	1.21	34	332	9.76

N = Total number of publications; C = total number of citations; C/D = cites per document.

**Table 7:** Micro topics (InCites).

Micro Topic	Code	N	C	C/D
Bibliometrics	6.238.166	4460	66,782	14.97
Knowledge Management	6.3.2	134	2199	16.41
Systematic Reviews	1.155.611	87	718	8.25
Corporate Social Responsibility	6.3.385	66	1113	16.86
Tourism	6.223.247	61	1014	16.62
Foresight	6.294.1807	39	689	17.67
Entrepreneurship	6.3.726	38	743	19.55
Environmental Kuznets Curve	6.115.234	31	471	15.19
Academic Entrepreneurship	6.3.1467	31	569	18.35
Information Literacy	4.48.228	30	153	5.10
Customer Satisfaction	6.3.65	29	369	12.72
Project Scheduling	4.224.599	28	495	17.68
Fuzzy Sets	4.61.56	28	857	30.61
Agglomeration Economies	6.86.280	27	356	13.19
Internationalization	6.3.1229	23	226	9.83
Internet of Things	4.13.807	22	481	21.86
Sentiment Analysis	4.48.672	21	149	7.10
Unified Health System	1.156.1509	20	106	5.30
Corporate Governance	6.10.63	20	379	18.95
Life Cycle Assessment	6.115.1181	20	258	12.90

N = Total number of publications; C = total number of citations; C/D = cites per document.

**Table 8:** Main indexes of WoS-JCR and Scopus-SJR bibliometric sources.

Rank	WoS—JCR						Scopus—SJR					
	Journal	N_1_	Cit_1_	Q_1_	IF_2_	IF_5_	Journal	N_2_	Cit_2_	Q_2_	IF_3_	CS
1	*Scientometrics*	1051	20,447	Q1	2.87	3.07	*Scientometrics*	1036	26,087	Q1	1.210	5.6
2	*Journal of Informetrics*	203	5691	Q1	4.61	4.41	*Library Philosophy and Practice*	307	406	Q2	0.220	0.3
3	*Sustainability*	180	852	Q2	2.58	2.8	*Journal of Informetrics*	204	7542	Q1	2.079	8.4
4	*Journal of the American Society for Information Science and Technology*	83	3178	n/a	n/a	n/a	*Sustainability*	185	1483	Q2	0.581	3.2
5	*Journal of the Association for Information Science and Technology*	81	1609	Q2	2.41	3.17	*Journal of the American Society for Information Science and Technology*	83	4018	N/A	N/A	N/A
6	*Revista Española de Documentación Científica*	74	252	Q3	1.3	1.12	*Revista Española de Documentación Científica*	81	552	Q2	0.497	1.7
7	*Journal of Cleaner Production*	74	1287	Q1	7.25	7.49	*Malaysian Journal of Library and Information Science*	81	732	Q2	0.414	1.3
8	*Current Science*	71	292	Q4	0.73	0.88	*Lecture Notes in Computer Science (including subseries Lecture Notes in Artificial Intelligence and Lecture Notes in Bioinformatics)*	78	244	Q2	0.427	1.9
9	*Research Evaluation*	64	914	Q2	2.57	3.41	*Espacios*	78	57	Q3	0.215	0.5
10	*Technological Forecasting and Social Change*	61	1934	Q1	5.85	5.18	*Journal of Cleaner Production*	75	1867	Q1	1.886	10.9
11	*Profesional de la Información*	60	351	Q3	1.58	1.42	*Journal of the Association for Information Science and Technology*	74	2317	Q1	1.270	7.9
12	*Environmental Science and Pollution Research*	59	352	Q2	3.06	3.31	*Current Science*	69	427	Q2	0.238	1.2
13	*International Journal of Environmental Research and Public Health*	52	167	Q1	2.85	3.13	*Research Evaluation*	67	1165	Q1	1.792	5.6
14	*PLOS ONE*	51	912	Q2	2.74	3.23	*ACM International Conference Proceeding Series*	64	114	N/A	0.200	0.8
15	*World Neurosurgery*	50	306	Q3	1.83	2.07	*Profesional de la Información*	62	615	Q1	0.480	2.1
16	*Malaysian Journal of Library and Information Science*	49	215	Q3	1.55	0.96	*Technological Forecasting and Social Change*	62	2662	Q1	1.815	8.7
17	*Investigación Bibliotecologica*	49	42	Q4	0.35	0.48	*CEUR Workshop Proceedings*	62	123	N/A	0.177	0.6
18	*Medicine*	49	245	Q3	1.55	2	*DESIDOC Journal of Library and Information Technology*	57	280	Q2	0.281	1.0
19	*Research Policy*	41	2541	Q1	5.35	7.93	*World Neurosurgery*	55	463	Q2	0.727	2.4
20	*Journal of Information Science*	35	645	Q2	2.41	2.34	*Environmental Science and Pollution Research*	55	403	Q2	0.788	4.9

N_1_ = Number of publications (WoS); Cit_1_ = Number of citations (WoS); Q_1_ = Quartile JCR (data 2019); IF_2_ = Journal Impact Factor JCR (data 2019); IF_5_ = 5-year Journal Impact Factor JCR (data 2019); N_2_ = Number of publications (Scopus); Cit_2_ = Number of citations (Scopus); Q_2_ = Quartile SJR (data 2019); IF_3_= Impact SJR (data 2019); CS = Cite Score (data 2019).

**Table 9:** Top 10 countries and affiliations publishing in *Scientometrics*.

Rank	Country	N	C	C/D	Affiliation	N	C	C/D
1	China	1174	7881	6.7	KU Leuven	271	4986	18.4
2	United States	1125	14,841	13.2	Magyar Tudomanyos Akademia	268	5660	21.1
3	Spain	693	7538	10.9	Leiden University	248	8665	34.9
4	United Kingdom	579	10,206	17.6	Consejo Superior de Investigaciones Científicas	210	2924	13.9
5	Netherlands	572	12,720	22.2	Universiteit Antwerpen	157	2642	16.8
6	Germany	558	7890	14.1	Wuhan University	152	1191	7.8
7	Belgium	469	7681	16.4	Universidad de Granada	132	1969	14.9
8	India	340	2787	8.2	Chinese Academy of Sciences	126	950	7.5
9	Hungary	315	5974	19.0	Dalian University of Technology	123	1375	11.2
10	Italy	314	3197	10.2	Indiana University Bloomington	122	1770	14.5

N = Number of publications (1978–2021); C = Number of citations (1978–2021); C/D = cites per document.

**Table 10:** CNCI (Category Normalized Citation Impact) from InCites and FWCI (Field-Weighted Citation Impact) from SciVal.

Rank	InCites					SciVal				
	WoS Journal Name	N	C	C/D	CNCI	Scopus Journal Name	N	C	C/D	FWCI
1	*Scientometrics*	1051	20,447	19.5	1.37	*Scientometrics*	1036	26,087	25.2	2.51
2	*Journal of Informetrics*	203	5691	28.0	2.02	*Library Philosophy and Practice*	307	406	1.3	0.61
3	*Sustainability*	180	852	4.7	0.89	*Journal of Informetrics*	204	7542	37.0	3.23
4	*Journal of the American Society for Information Science and Technology*	83	3178	38.3	5.14	*Sustainability*	185	1483	8.0	1.54
5	*Journal of the Association for Information Science and Technology*	81	1609	19.9	7.27	*Journal of the American Society for Information Science and Technology*	83	4018	48.4	2.19
6	*Revista Española de Documentación Científica*	74	252	3.4	0.3	*Revista Española de Documentación Científica*	81	552	6.8	0.94
6	*Journal of Cleaner Production*	74	1287	17.4	1.27	*Malaysian Journal of Library and Information Science*	81	732	9.0	0.72
8	*Current Science*	71	292	4.1	0.42	*Lecture Notes in Computer Science*	78	244	3.1	0.91
9	*Research Evaluation*	64	914	14.3	3.4	*Espacios*	78	57	0.7	0.11
10	*Technological Forecasting and Social Change*	61	1934	31.7	2.39	*Journal of Cleaner Production*	75	1867	24.9	2.35
11	*Profesional de la Información*	60	351	5.9	1.67	*Journal of the Association for Information Science and Technology*	74	2317	31.3	2.83
12	*Environmental Science and Pollution Research*	59	352	6.0	0.99	*Current Science*	69	427	6.2	0.25
13	*International Journal of Environmental Research and Public Health*	52	167	3.2	1.44	*Research Evaluation*	67	1165	17.4	1.75
14	*PLOS ONE*	51	912	17.9	1.61	*ACM International Conference Proceeding Series*	64	114	1.8	0.39
15	*World Neurosurgery*	50	306	6.1	1.43	*Profesional de la Información*	62	615	9.9	2.90
16	*Malaysian Journal of Library and Information Science*	49	215	4.4	0.35	*Technological Forecasting and Social Change*	62	2662	42.9	3.95
16	*Investigación Bibliotecológica*	49	42	0.9	0.11	*CEUR Workshop Proceedings*	62	123	2.0	0.71
16	*Medicine*	49	245	5.0	0.78	*DESIDOC Journal of Library and Information Technology*	57	280	4.9	0.76
19	*Research Policy*	41	2541	62.0	2.73	*World Neurosurgery*	55	463	8.4	1.25
20	*Journal of Information Science*	35	645	18.4	1.13	*Environmental Science and Pollution Research*	55	403		1.38

**Table 11:** Top 10 countries and affiliations publishing in Medicine category.

Rank	Country	N	Affiliation (Country)	N
1	United States	1919	University of Valencia (Spain)	110
2	China	834	University of Toronto (Canada)	110
3	United Kingdom	688	Harvard Medical School (USA)	102
4	Spain	597	Universidade de Sao Paulo—USP (Brasil)	93
5	Canada	458	McMaster University (Canada)	86
6	Brazil	359	Consejo Superior de Investigaciones Científicas (Spain)	80
7	Australia	336	Universidad Miguel Hernandez de Elche (Spain)	73
8	Germany	303	The University of Sydney (Australia)	67
9	France	226	An-Najah National University (Palestine)	61
10	Italy	223	The University of British Columbia (Canada)	56

N = Number of publications (1978–2021); C = Number of citations (1978–2021); C/D = cites per document.

**Table 12:** Top 10 medical keywords in bibliometric publications in this category and the main affiliations using them.

Medicine Topic	N	Main Affiliation (Country)
Epidemiology	194	Universidad Tecnológica de Pereira (Colombia)
Pediatrics	194	University of Valencia (Spain)
Orthopedics	186	Centre Hospitalier Universitaire de Clermont-Ferrand (France) CNRS Centre National de la Recherche Scientifique (France) Second Military Medical University (China) McMaster University (Canada)
Cardiology	166	Universidade de Sao Paulo—USP (Brasil)
Neurosurgery	164	University of Tennessee Health Science Center (USA)
Radiology	152	Hallym University, College of Medicine (South Korea)
Ophthalmology	134	China Medical University Shenyang (China)
Oncology	131	University of Texas MD Anderson Cancer Center (USA) University of Michigan, Ann Arbor (USA)
Plastic Surgery	121	Harvard Medical School (USA) Massachusetts General Hospital (USA)
Psychiatry	119	King’s College London (UK) Universidad de Alcalá (Spain)

**Table 13:** Top 10 journals publishing articles on bibliometrics in the category of medicine and their main bibliometric source indices.

Rank			WoS—JCR			Scopus—SJR		
	Journal	N_1_	Q_1_	IF_2_	IF_5_	Q_2_	IF_3_	CS
1	*Journal Of The Medical Library Association*	87	Q2	2.042	2.299	Q1	0.894	2.8
2	*International Journal Of Environmental Research And Public Health*	83	Q1	2.849	3.127	Q2	0.739	3.0
3	*World Neurosurgery*	82	Q3	1.829	2.074	Q2	0.727	2.4
4	*Journal Of Clinical Epidemiology*	55	Q1	4.952	6.234	Q1	2.702	9.0
5	*BMJ Open*	42	Q2	2.496	2.992	Q1	1.247	3.5
6	*Health Research Policy And Systems*	40	Q2	2.365	2.762	Q1	0.987	3.8
7	*Medicine United States*	40	Q3	1.552	1.998	Q2	0.639	2.7
8	*Plastic And Reconstructive Surgery*	37	Q1	4.235	4.387	Q1	1.916	5.3
9	*Revista Cubana De Informacion En Ciencias De La Salud*	36	n/a	n/a	n/a	Q3	0.172	0.5
10	*Health Information And Libraries Journal*	35	Q3	1.356	1.280	Q2	0.521	2.6

N_1_ = Number of publications (Scopus); Q_1_ = Quartile JCR (data 2019); IF_2_ = Journal Impact Factor JCR (data 2019); IF_5_ = 5-year Journal Impact Factor JCR (data 2019); Q_2_ = Quartile SJR (data 2019); IF_3_ = Impact SJR (data 2019); CS = Cite Score (data 2019).

**Table 14:** Top 10 countries and affiliations publishing in Environmental Sciences category.

Rank	Country/Region	N	Affiliation (Country)	N
1	China	485	Chinese Academy of Sciences (China)	94
2	Spain	191	Universidad de Almeria (Spain)	47
3	United States	177	Asia University Taiwan (China)	38
4	Brazil	122	University of Chinese Academy of Sciences (China)	30
5	United Kingdom	113	Beijing Institute of Technology (China)	29
6	Australia	81	Peking University (China)	27
7	Italy	75	Ministry of Education China (China)	25
8	Germany	56	Research Center for Eco-Environmental Sciences Chinese Academy of Sciences (China)	19
9	Canada	54	University of Valencia (Spain)	18
10	Taiwan	50	Tianjin University (China) Beijing Normal University (China) Wuhan University (China)	18

N = Number of publications (1978–2021); C = Number of citations (1978–2021); C/D = cites per document.

**Table 15:** Top 10 environmental sciences keywords in bibliometric publications in this category and the main affiliations using them.

Environmental Sciences Topic	N	Main Affiliation (Country)
Sustainability	214	Universidad de Almeria (Spain)
Sustainable Development	207	Universidad de Almeria (Spain)
Climate Change	144	Chinese Academy of Sciences (China)
Ecology	66	Chinese Academy of Sciences (China)
Environmental Impact	58	Universidad de Almeria (Spain)
Biodiversity	57	Chinese Academy of Sciences (China)
Environmental Protection	45	Chinese Academy of Sciences (China)
Environmental Management	44	Chinese Academy of Sciences (China)
Public Health	43	Goethe-Universität Frankfurt am Main (Germany)
Environmental Monitoring	37	Chinese Academy of Sciences (China)

**Table 16:** Top 10 journals publishing articles on bibliometrics in the category of environmental sciences and their main bibliometric source indices.

Rank			WoS—JCR			Scopus—SJR		
	Journal	N_1_	Q_1_	IF_2_	IF_5_	Q_2_	IF_3_	CS
1	*Sustainability Switzerland*	239	Q2	2.576	2.798	Q2	0.581	3.2
2	*Journal Of Cleaner Production*	108	Q1	7.246	7.491	Q1	1.886	10.9
3	*International Journal Of Environmental Research And Public Health*	83	Q1	2.849	3.127	Q2	0.739	3.0
4	*Environmental Science And Pollution Research*	60	Q2	3.056	3.306	Q2	0.788	4.9
5	*Science Of The Total Environment*	30	Q1	6.551	6.419	Q1	1.661	8.6
6	*Acta Ecologica Sinica*	30	n/a	n/a	n/a	Q3	0.229	1.1
7	*Science And Public Policy*	26	Q3	1.730	2.114	Q1	0.771	3.3
8	*Water Switzerland*	22	Q2	2.544	2.709	Q1	0.657	3.0
9	*IOP Conference Series Earth And Environmental Science*	19	n/a	n/a	n/a	Q3	0.175	0.4
10	*Ecological Indicators*	15	Q1	4.229	4.968	Q1	1.331	7.6

N_1_ = Number of publications (Scopus); Q_1_ = Quartile JCR (data 2019); IF_2_ = Journal Impact Factor JCR (data 2019); IF_5_ = 5-year Journal Impact Factor JCR (data 2019); Q_2_ = Quartile SJR (data 2019); IF_3_ = Impact SJR (data 2019); CS = Cite Score (data 2019).

**Table 17:** Main keywords of each cluster.

Cluster 5	Cluster 4	Cluster 1	Cluster 3	Cluster 6	Cluster 2	Cluster 0
Science Mapping	Research Productivity	Medicine	Environmental Sciences	Psychology	Nursing	Engineering
28.72%	23.29%	19.65%	11.84%	7.02%	5.66%	3.82%
Vosviewer	Citation Analysis	Citation Analysis	Research Trends	Bibliometric Indicators	Citation Analysis	Nanotechnology
Citation Analysis	H-index	Citations	Web Of Science	Impact Factor	Authorship Pattern	Scientometrics
Web Of Science	Citations	Publications	Scientometrics	Bibliometry	Scientometrics	Citation Analysis
Literature Review	Research Evaluation	Scientometrics	Citespace	Spain	Nursing	Text Mining
Scopus	Scientometrics	H-index	Sci-expanded	Research	Research Productivity	Information Retrieval
Scientometrics	Impact Factor	Impact Factor	Social Network Analysis	Scientific Journals	Bibliometric Study	Patent Analysis
Bibliometric Study	Altmetrics	Research	Scopus	Scientometrics	Scopus	Digital Libraries
Co-word Analysis	Web Of Science	Web Of Science	Citations	Journals	India	Citations
Sustainability	Scopus	Scopus	Citation Analysis	Bibliometric Study	Author Productivity	Nanoscience
Co-citation Analysis	Peer Review	Journal Impact Factor	Climate Change	Publications	Nursing Research	China
Network Analysis	Journal Impact Factor	Pubmed	Publications	Citations	Citations	Citation Network
Science Mapping	Italy	Bibliometric Study	Impact Factor	Web Of Science	Impact Factor	Technology Forecasting
Social Network Analysis	Research Assessment	Vosviewer	Sci	Psychology	Library And Information Science	Computational Linguistics
Citations	Publications	COVID-19	Research	Databases	Lotka’s Law	Emerging Technologies
Content Analysis	Research	Research Productivity	Vosviewer	Periodicals	Research Output	Research Evaluation
Citespace	Google Scholar	Biomedical Research	Sustainability	Scopus	Degree Of Collaboration	Document Clustering
Co-citation	Universities	Latin America	H-index	Citation Analysis	Bibliometric Indicators	Bibliometric Study
Research Trends	Research Productivity	Citespace	Scientific Production	Journal Article	Scientific Production	Publications
Bibliometric Review	Evaluation	Bibliometric Indicators	Research Hotspots	Impact	Research	Network Analysis

**Table 18:** Main database used for each cluster.

Cluster	Name	WoS	Scopus	Main Country keyword
Cluster 5	Science Mapping	192	133	China
Cluster 4	Research Productivity	73	61	Italy
Cluster 1	Medical Research	81	75	China/India
Cluster 3	Environment	102	37	China
Cluster 6	Psychology	22	17	Spain
Cluster 2	Nursing	12	20	India
Cluster 0	Engineering	2	1	China

## Data Availability

Data retrieved from Scopus, SciVal, WoS, Incites, JCR and SJR databases.
